# Chado use case: storing genomic, genetic and breeding data of Rosaceae and Gossypium crops in Chado

**DOI:** 10.1093/database/baw010

**Published:** 2016-03-17

**Authors:** Sook Jung, Taein Lee, Stephen Ficklin, Jing Yu, Chun-Huai Cheng, Dorrie Main

**Affiliations:** Department of Horticulture, Washington State University Pullman, WA, USA

## Abstract

The Genome Database for Rosaceae (GDR) and CottonGen are comprehensive online data repositories that provide access to integrated genomic, genetic and breeding data through search, visualization and analysis tools for Rosaceae crops and *Gossypium* (cotton). These online databases use Chado, an open-source, generic and ontology-driven database schema for biological data, as the primary data storage platform. Chado is highly normalized and uses ontologies to indicate the ‘types’ of data. Therefore, Chado is flexible such that it has been used to house genomic, genetic and breeding data for GDR and CottonGen. These data include whole genome sequence and annotation, transcripts, molecular markers, genetic maps, Quantitative Trait Loci, Mendelian Trait Loci, traits, germplasm, pedigrees, large scale phenotypic and genotypic data, ontologies and publications. We provide information about how to store these types of data in Chado using GDR and CottonGen as examples sites that were converted from an older legacy infrastructure.

**Database URL:** GDR (www.rosaceae.org), CottonGen (www.cottongen.org)

## Introduction

The database schema Chado (1) is a part of GMOD, the Generic Model Organism Database project (www.gmod.org), a collection of open source software tools for managing, visualizing, storing and disseminating genetic and genomic data. There are >40 software projects in GMOD, providing functionality such as genome visualization and editing, database tools, comparative genome visualization, genome annotation, community annotation and gene expression visualization. Commonly used GMOD software includes GBrowse (2), JBrowse (3), CMap (4), Pathway Tools (5), GBrowse_syn (6), Sybil (7), Apollo (8), BioMart (9), InterMine (10) and Tripal (11, 12).

Chado is the only database schema in GMOD. It was originally developed for the FlyBase database to integrate *Drosophila* genomic sequences and annotation data with genetic and phenotypic data (1). The schema was designed to be generic, extensible and open-source, so it could be used to build databases for any model organism with widely different metadata. The current version of Chado, version 1.2, contains > 400 tables for storing a variety of biological data, and improvements and updates to Chado occur through a community-involved open process.

Chado extensively uses controlled vocabularies and ontologies to describe data, properties of data and relationships between data. For example, the Sequence Ontology (SO) (13) is a commonly used vocabulary to describe genomic sequences and their annotation. Genomic sequence data, such as genes or genetic markers are identified in Chado using the corresponding terms from the SO. Relationships between data in Chado are also described using controlled vocabularies. Terms such as ‘is_a’ and ‘part_of’, allow for relationships to be defined. The SO contains relationship terms, but also the Relationship Ontology (RO) (https://gist.githubusercontent.com/scottcain/10e255c991a41bcf0,187/raw/7faba8c6f26 766f5a686eb681f5cb2f48e49b78a/ro.obo) contains terms for creating relationships between data. For example, hierarchical entities of genomic features (gene, CDS and so forth) are stored in the Chado table named ‘feature,’ and the relationships for these data are defined in the ‘feature_relationship’ table. The usage of controlled vocabularies stored in the cvterm table, instead of field names, gives flexibility but it requires rather complex queries to retrieve data. Materialized views can be created, however, for simpler and faster queries.

Another important characteristic of Chado is its tables are organized into groups called modules, each responsible for different data domains. Example modules include the sequence, phenotype, genetic, controlled vocabulary, publication and phylogeny modules to name a few (http://gmod.org/wiki/Chado). The modular schema allows developers to select only the groups of tables needed to store their data. There are a few tables that are ‘core’ to all others (such as those housing vocabularies) and are required by all Chado installations. The modular nature of Chado allows creation of new modules when sufficient need arises.

Chado is increasingly used to build online genome databases due to the growing number of large-scale sequencing projects and the need for these datasets to be published in a searchable online format. The availability of original publication of Chado (1), as well as the wiki document on the Chado Sequence Module (http://gmod.org/wiki/Chado_Sequence_Module) that provide principles and examples in describing and storing genomic sequences in Chado has been useful in building these genome databases. Some examples of genome databases that use Chado include the Hardwood Genomics Project (http://www.hardwoodgenomics.org/) (14), the Banana Genome Hub (http://banana-genome.cirad.fr/) (15), the Medicago truncatula genome database (http://medicago.jcvi.org/MTGD/?q=home) (16), the i5k Workspace (https://i5k.nal.usda.gov/) (17) and the Arabidopsis Information Portal (https://www.araport.org).’

The genetic and phenotype modules of Chado enable storage of genotype, phenotype and their relationships, however, they do not support data from multiple large-scale phenotypic and genotypic projects. To meet this need, a Natural Diversity (ND) module was added to Chado through collaborative efforts by a consortium of representatives from several online genome database projects (18). Chado, with the addition of the new ND module, now allows for storage of data from experimental lines that are scored for a large number of phenotypic traits at multiple times, in multiple environmental conditions, and genotyped with a set of genetic markers. In addition to storing data from experiments performed on existing lines, experiments that generate new lines and experimental samples, such as field collections, crosses and treatments, can be stored using the ND module. Developers of the Solanceae Genomics Network database (SGN, http://solgenomics.net/) (19), VectorBase (http://www.vectorbase.org/) (20) and Knowpulse: Pulse Crop Genomics & Breeding (http://knowpulse2.usask.ca/portal) (21), in addition to the Genome Database for Rosaceae (GDR) (22–24), are some of the participants who designed the ND module to store their large scale genotypic and phenotypic data. Their use case to store large scale phenotypic and genotypic data in the ND module are available from Jung *et al.* (18). SGN uses Chado to store genomic data and large scale genotypic and phenotypic data in conjunction with in-house schema that store other types of data.

Many of the Chado-based genomic databases (14–17) are built using Tripal, a web front end for Chado, which integrates with the popular Drupal (http://www.drupal.org) Content Management System (CMS). Tripal is designed to decrease the cost and time required to publish data housed in Chado in an online searchable format. It also provides an Application Program Interface (API) that allows for complete customization of data should site developers require changes to the default presentation. As a result, site developers are able to exchange code they develop for their sites with other Tripal users and exchange information about use cases and best practices for data storage in Chado. The availability of Tripal significantly increased the adoption of Chado as a database schema for genomic databases. CassavaBase (http://www.cassavabase.org/) and VectorBase (20) is example databases built using Chado, but not Tripal.

The Genome Database for Rosaceae (GDR) and CottonGen are (25) one of the first, if not the only, databases that adopted Chado/Tripal solely to store integrated genetic, genomic and breeding data comprised of genes, transcripts, genome annotation, genetic maps, markers, QTLs, Mendelian trait loci (MTL), germplasm, pedigree and large-scale phenotypic and genotypic data from breeding, cultivar evaluation and conservation projects. The data are stored in Chado in similar ways in the following databases developed by the GDR and CottonGen team: the Citrus Genome Database (http://www.citrusgenomedb.org) (26) and the Cool Season Food Legume Database (http://www.coolseasonfoodlegume.org) (27). Some Chado and Tripal based databases also include databases with genomic and genetic data, such as the Legume Information System (http://legumeinfo.org/) and PeanutBase (http://peanutbase.org/), and genome databases with resources for breeders such as Knowpulse (21).

GDR is a comprehensive online database resource for basic, translational and applied Rosaceae researchers (22–24). The Rosaceae family includes many crops of economic and nutritional importance such as almond, apple, apricot, blackberry, cherry, peach, pear, plum, raspberry, rose and strawberry. Initiated in 2003, GDR began with transcriptome and genetic data, stored in a custom-developed schema. With the advent of whole genome sequences annotation data and large-scale phenotypic and genotypic data in Rosaceae, the data in GDR was migrated from a legacy database schema to Chado. CottonGen (25) is a curated and integrated online database for cotton. CottonGen supercedes CottonDB (28) and the Cotton Marker Database (CMD) (29), with enhanced tools for data sharing, mining, visualization and data retrieval of cotton research data. CottonDB, founded in 1995, had a hybrid database system; the genomic, genetic, taxonomic and bibliographic data were stored in an object-oriented AceDB database (30), while the genetic maps and genome sequences were maintained in a MySQL relational database. CMD used a custom MySQL database schema. CottonGen stores the original data from CottonDB and CMD, as well as newly obtained data including whole genome sequences and annotation data, in Chado.

The purpose of this report is to provide practical examples of how the genomic, genetic and breeding data are stored and integrated in Chado for GDR and CottonGEN, and to show how legacy database schemas can be converted into Chado. The following sections describe how GDR and CottonGen use Chado to store genome, genetic, germplasm, phenotype, and genotype data in Chado. In these sections, we describe the data types, the vocabularies they are mapped to and the Chado tables and fields in which they are stored. For brevity, we do not indicate every value for every field of every table in Chado that we use. But we do provide the necessary fields and terms to duplicate our storage methods. To determine appropriate values for unmentioned table fields, readers are referred to the Chado schema documentation to infer appropriate values—which typically are easy to determine given the context for the data. When referring to fields in Chado, we use the convention [table].[field] where [table] represents the Chado table name and [field] is the name of a field within that table (e.g. feature.type_id, featurprop.type_id, feature_relationship.type_id). When in context, the field may be referred to simply by the field name without the table prefix. A high-level entity-relationship (ER) diagram is provided in Supplementary File S1.

## Genomic data

**Figure 1:**
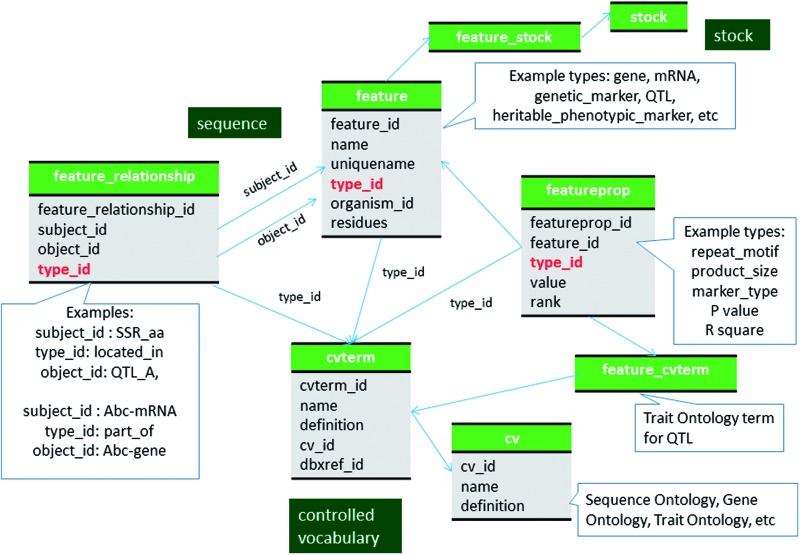
Schematic diagram of how genomic features are stored in Chado using ontology. The bold red fields represent foreign keys to the cvterm table which houses vocabulary terms. Boxes in dark green represents the modules of Chado represented in this diagram.

Chado has been widely used to store genome-associated biological information for a range of organisms (16–17). The genomic data in GDR and CottonGEN are stored using the sequence module of Chado following the description in Mungall *et al.* (1) and the documentation on the GMOD website (http://gmod.org/wiki/Chado_Best_Practices). For most genomic data which is often made available in the common GFF3 format, the Tripal and Perl-based Chado loaders will import data following these recommendations. Therefore, we do not provide an in depth discussion for how these data are stored in Chado, except to state that for GDR and CottonGen, all genomic features such as chromosomes, genes, mRNA, ncRNA, CDS, 5′ UTRs, 3′ UTRs and polypeptides, to name a few, are stored in the feature table of Chado with association to appropriate SO terms in the feature.type_id field (Figure 1). The relationships between features, such as between a gene, mRNA, CDS and proteins, are stored in the feature_relationship table, using SO relationship terms (e.g. ‘part_of’ and ‘derives_from’) in the feature_relationship.type_id field. Whenever possible, the locations of the features when localized to another are stored in the featureloc table.

GDR and CottonGen do store other genomic data which is not often available in GFF3 format. For these data we follow a similar storage method. For example, Expressed Sequence Tags (ESTs) downloaded from NCBI dbEST (31) and EST unigene contigs created in-house or shared by collaborators are also stored in the feature table with annotations housed in the feature_cvterm table. Additionally, both GDR and CottonGen integrate gene data directly from NCBI. An in-house Perl script is used to automatically download species-specific gene data and import that into Chado. These nucleotide sequences, genes, mRNA, CDS, 5′UTRs and 3′UTRs, are parsed and each is stored in the feature table. The gene symbols, also parsed from NCBI data (eg. NIP6.1), are stored as an additional separate record in the feature table to serve as the ‘reference’ gene. The reference genes are in turn associated with each instance of a gene via the feature_relationship table using SO term ‘associated_with’ in the feature_relationship.type_id field. Thus, the reference gene does not contain sequence residues in the feature.residues field, but genes that represent instances of the gene do have residues. The reference genes belong to an analysis, which is stored in the analysis table and linked to the feature table by the analysisfeature table.

## Genetic data

### Molecular markers and genetic maps

**Figure 2:**
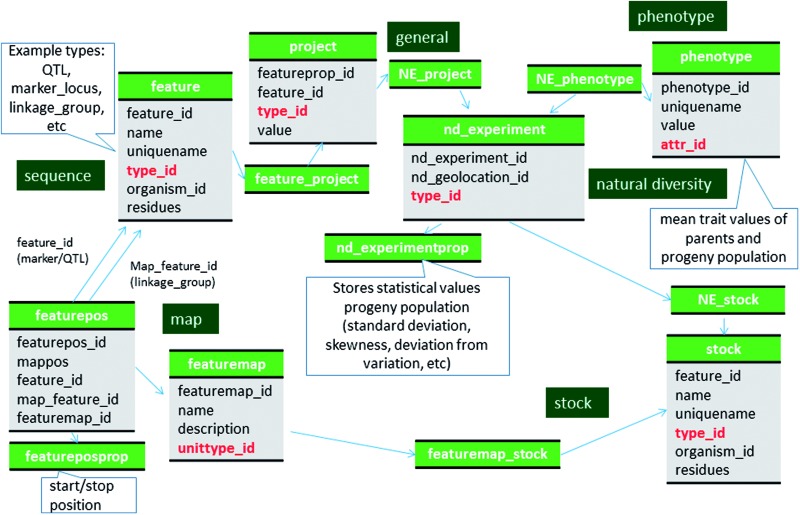
Schematic diagram of how the genetic map data of molecular markers and QTL are stored in Chado. The bold red fields represent foreign keys to the cvterm table which houses vocabulary terms. Boxes in dark green represents the modules of Chado represented in this diagram.

Molecular markers, such as SSR, RFLP, SNPs, etc, are stored in the feature table with the SO term ‘genetic_marker’ in the feature.type_id field (Figure 2, Table 1; Supplementary File S1). The specific marker type, such as SSR, RFLP and SNP, is stored in the featureprop table with a term ‘marker_type’ in the featureprop.type_id field. Details of the molecular marker such as PCR condition and restriction enzyme are also stored in the featureprop table (Table 1). The source germplasm of the marker is stored in the stock and linked by the feature_stock table with ‘source’ as the term for feature_stock.type_id.

**Table 1.** Storage of Genetic markers in Chado. Genetic markers are stored in feature table with a type_id of 'genetic_marker'

Data type	Chado module^a^	Table name	Field name	Vocabulary terms for type_id	Vocabulary^b^	Description
Marker name	Sequence	feature	uniquename			The unique name of the marker
Source organism	Sequence	Feature	organism_id			The organism to which this marker belongs. A foreign key to the organism table.
Type	Sequence	Feature	type_id	genetic_marker	SO	All markers are of the SO type: 'genetic marker'.
Properties of a genetic marker						
Data type	Chado module^a^	Table name	Field name	Vocabulary terms for type_id	Vocabulary^b^	Description

Alias	Sequence	Featureprop	Value	alias	In-house	A synonym or alias of the marker.
Marker type	Sequence	Featureprop	Value	marker_type	In-house	The actual marker type such as SSR, SNP, RFLP, etc.
Repeat motif	Sequence	featureprop	Value	repeat_motif	In-house	For SSR markers a repeat motif is stored.
Restriction enzyme	Sequence	featureprop	Value	restriction_enzyme	In-house	Restriction enzymes for the restriction site associated markers, such as RFLP, AFLP, etc.
Product length	Sequence	Featureprop	Value	product_length	In-house	The product size of the PCR-based markers such as SSR.
Maximum length	Sequence	Featureprop	Value	max_length	In-house	Maximum length of the PCR products observed in the original study that developed the marker.
minimum length	Sequence	Featureprop	Value	min_length	In-house	Minimum length of the PCR products observed in the original study that developed the marker.
is codonimant	Sequence	Featureprop	Value	is_codominant	In-house	whether the marker is codominant or not.
PCR condition	Sequence	Featureprop	Value	PCR_condition	In-house	Thermocycling condition of the the PCR protocol for PCR-based markers.
Screening method	Sequence	Featureprop	value	screening_method	In-house	gel type, % etc (eg. 2% agarose) for electrophoresis of PCR product and any other screening methods for other types of markers
Comments	Sequence	Featureprop	value	comments	In-house	Additional comments about the genetic marker.
Source description	Sequence	Featureprop	value	source	In-house	Whether the marker was developed from the sequence of EST, BAC, cDNA, genomic clone, or whole genome sequencing.
Alleles	Sequence	Featureprop	value	allele	SO	The marker alleles. Separated with a forward slash '/' character.
5' flanking seq	Sequence	Featureprop	value	five_prime_flanking_region	SO	The 5' flanking sequence of the marker.
3' flanking seq	Sequence	Featureprop	value	three_prime_flanking_region	SO	The 3' flanking sequence of the marker.
Other data linked to a genetic marker						
Data Type	Chado Module^a^	Table Name	Linking Table^c^	Description		

Source germplasm	Stock	Stock	feature_stock*	The germplasm, from which the marker was developed, has a record in the stock table and is associated with the marker.		
Contact	Contact	Contact	feature_contact*	The individual that submitted the marker has a record in the contact table and is associated with the marker.		
Reference	Publication	pub	feature_pub	Associates a publication stored in the pub table with the genetic markerl		

Data Type	Chado Module^a^	Table Name	Linking Table^c^	Description		

Primer	Sequence	Feature	feature_relationship	The primer is a separate record in the feature table (with uniquename as [marker name].[primer name] with the sequence stored in feature.residue, type_id is SO:primer). The relationship type_id is 'adjacent_to' and the primer is the subject_id and marker is the object_id.		
Probe	Sequence	Feature	feature_relationship	The probe is a separate record in the feature table (with type_id as SO:probe). The relationship type_id 'associated_with', the probe is the subject_id and the marker is the object_id.		
Source sequence	Sequence	Feature	feature_relationship	The source sequence from which the marker is developed is stored in the feature table (with type_id as SO:sequence_feature). The relationship type_id is 'derived_from', the marker is the subject_id and the sequence is the object_id.		
Marker locus	Sequence	Feature	feature_relationship	When marker is localized in genomic sequence, the locus is stored separately in the feature table (with type_id as SO:marker_locus). The relationship type_id is 'instance_of', the subject_id is the locus and the object_id is the marker.		
GBID	General	dbxref	feature_dbxref	The GenBank ID accession is associated with the marker.		
dbSNP_ID	General	dbxref	feature_dbxref	The dbSNP Id is associated with SNP markers.		

a The Chado modules are for Chado version 1.2.

b The vocabularies are: Sequence Ontology (SO), In-House (term add to the GDR/CottonGen internal vocabularies).

c Tables with an asterisk (*) are custom tables.

The SNP allele (eg. A/T), five prime flanking sequence and three prime flanking sequences are stored in the featureprop table with type_id of ‘allele’, ‘five_prime_flanking_region’ and ‘three_prime_flanking_region’, respectively. The SNP alleles are converted to IUPAC code and concatenated with five prime and three prime flanking sequences before we store in the feature.residue field. This way, allele data as well as the entire sequence data that can be used in sequence analysis tools can be provided to users. Sequences of other types of markers (e.g. sequence of PCR products for PCR-based markers), are also stored in feature.residue. If a marker is developed from a known sequences (e.g. sequence of BAC clone, cDNA clone, etc), the sequence is stored as a new record in the feature table with the SO term ‘sequence_feature’ in the feature.type_id field. The relationship to the marker and the sequence is stored in the feature_relationship table with a SO term ‘sequence_of’ in the feature_relationships.type_id field.

Genetic marker primers are stored in the feature table with the SO term ‘primer’ in the feature.type_id field and their relationship is stored in the feature_relationship table with the SO term ‘adjacent_to’ in the feature.type_id field. The probes of SNP markers are also stored as a feature with type_id ‘probe’ and the relationship with the marker is stored in the feature_relationship table with type_id ‘associated_with’. When the marker is also available in NCBI (e.g. dbSNP), the NCBI accession number is stored in the dbxref table and associated with the genetic marker record via the feature_dbxref table.

When the gel image of the PCR-based markers are available, we store the image in the eimage table and link to the feature table using the feature_image table (Supplementary File S1). The name of the file is stored in the eimage.image_url field. The title and description of the image file is stored in the eimageprop table with the type_id for ‘title’ and ‘description’, respectively. The person who provided the image is stored in the contact table and linked to the eimage table using the eimage_contact table.

Locus names of molecular markers that have been mapped to genetic maps are stored in the feature table with type_id as the SO term ‘marker_locus’ and the relationship between the marker is stored in the feature_relationship table with type_id as the RO term ‘instance_of’. Locus names are usually the same as marker names but when the same marker is mapped to more than one position in the same genetic map, distinct locus names are associated with each position in the map. A feature of SO term ‘marker_locus’ is therefore associated with the genetic map position (Figure 2). Linkage groups stored in the feature table with SO term ‘linkage_group’ as the type_id. Genetic maps are stored in the featuremap table (Table 2). The associated genetic map, linkage group and locus (marker_locus, QTL or bin) are stored in the featurepos table (Table 3). We store map positions (cM) in the featureposprop table, not in the featurepos.mappos field, since most QTL have three associated positions, start, stop and peak (Table 3). The relationship between the locus and bin is stored in feature_relationship table using 'located_in' as the type_id. The mapping population is stored in the stock table and linked to the featuremap table via the featuremap_stock (Figure 2).

**Table 2.** Storage of Genetic map data in Chado. Genetic maps are stored in featuremap table

Data type	Chado module^a^	Table name	Field name	Vocabulary terms for type_id	Vocabulary^b^	Description
Map name	Map	featuremap	name			Name of the genetic map
Map unit	Map	featuremap	unittype_id	cM, bin_unit	In-house	Units of the genetic map
Properties of a genetic map						
Data type	Chado module^a^	Table name	Field name	Vocabulary terms for type_id	Vocabulary^b^	Description

Map type	Map	featuremapprop*	type_id	map_type	In-house	Map type such as genetic linkage map, in silico map or association map.
Analysis method	Map	featuremapprop*	type_id	analysis_method	In-house	Any analysis method that is used to build the map.
Software	Map	featuremapprop*	type_id	software	In-house	Any software that is used to build the map such as MapMaker.
Comments	Map	featuremapprop*	type_id	comments	In-house	Any comments about the map.
Genome group	Map	featuremapprop*	type_id	genome_group	In-house	Cotton specific data: the genome groups (one of the eight groups of diploid cotton) that the map corresponds to.
Population type	Map	featuremapprop*	type_id	population_type	In-house	Type of the mapping population such as F1, F2, BC1.
Other data linked to a genetic marker						
Data type	Chado module^a^	Table name	Linking table^c^	Description		

Population	Stock	stock	featuremap_stock*	Associates the mapping population stored in the stock table.		
Contact	General	contact	featuremap_contact*	Associates the contact information stored in the contact table.		
Organism	Organism	organism	featuremap_organism*	Associates the species information of the genetic map stored in the organism table.		
Reference	Pub	pub	featuremap_pub*	Associates the publication stored in the pub table.		

a The Chado modules are for Chado version 1.2.

b The vocabularies are: Sequence Ontology (SO), In-House (term add to the GDR/CottonGen internal vocabularies).

c Tables with an asterisk (*) are custom tables.

**Table 3.** Storing positions of genetic markers and trait loci in genetic maps in Chado. The map position data are stored in the featurepos and featureposprop tables

Data type	Chado module^a^	Table name^c^	Field name	Vocabulary terms for type_id	Vocabulary^b^	Description
locus name	Map	featurepos	feature_id			A foreign key to the feature table. Refers to the features with type 'marker_locus', 'QTL', 'heritable_morphological_marker' or 'bin'.
map name	Map	featurepos	featuremap_id			A foreign key to the featuremap table. Refers to the genetic map.
linkage group	Map	featurepos	map_feature_id			A foreign key to the feature table. Refers to the features with type 'linkage_group'.
start	Map	featureposprop*	Value	start	In-house	The start position of the marker, QTL, or bin in the linkage group
stop	Map	featureposprop*	Value	stop	In-house	The stop position of the marker, QTL, or bin in the linkage group
QTL peak	Map	featureposprop*	Value	qtl_peak	In-house	The peak position of QTL.
probability	Map	featureposprop*	Value	probability	In-house	probability of the QTL span
comments	Map	featureposprop*	Value	comments	In-house	Any comments on the map position data.

a The Chado modules are for Chado version 1.2.

b The vocabularies are: Sequence Ontology (SO), In-House (term add to the GDR/CottonGen internal vocabularies).

c Tables with an asterisk (*) are custom tables.

### Qtl

QTL are stored in the feature table with a SO term of ‘QTL’ as the type_id (Figure 1, Table 4, Supplementary File S1). Curator-given names, unique for each QTL reported in publications, are stored in the feature.uniquename field. QTL aliases are stored in the feature_synonym table. The Trait Ontology (TO) (32) is used to specify Trait names for QTLs. Chado expects that in-house terms may be needed when community-defined ontologies are insufficient. Therefore, if an appropriate term is not available in TO, we add new terms to a special in-house vocabulary. The TO, plus any newly added terms constitutes the ‘Rosaceae Trait Ontology’ and the ‘Cotton Trait Ontology’ for GDR and CottonGen respectively. We submit the new terms to TO for review with the hope of official inclusion. The association of QTL with traits (TO terms) is made via the feature_cvterm table. Statistical values such as *R*-square, LOD, additive effect, dominance effect and other associated data such as published symbols, screening methods and comments are stored in the featureprop table (Figure 1, Table 4). The relationship with the colocalized markers and neighboring markers are stored in the feature_relationship table with RO terms of ‘located_in’ and ‘adjacent_to’ used as types respectively (Figure 1). QTLs from the same study are associated via a record in the project table using the feature_project table (Figure 2). The positions of QTLs in genetic maps are stored the same way as those of markers (Figure 2). If the QTLs are anchored to the genome, the position is stored in the featureloc table. The mapping population of QTL maps are stored in the stock table and linked through the featuremap_stock (Figure 2). The parent germplasm which is responsible for the desirable trait is associated with the QTL feature via the feature_stock table.

**Table 4.** Storage of QTL in Chado. QTL are stored in feature table with a type_id of 'QTL'

Data type	Chado module^a^	Table name	field name	Vocabulary terms for type_id	Vocabulary^b^	Description
QTL label	Sequence	feature	Uniquename			curator-defined label for QTL
organism	Sequence	feature	organism_id			The organism to which the QTL belongs. A foreign key to the organism table.
type	Sequence	feature	type_id	QTL	SO	All QTL are of the SO type: 'QTL'.
Properties of QTL						
Data Type	Chado Module^a^	Table Name	Field Name	Vocabulary terms for type_id	Vocabulary^b^	Description

Published symbol	Sequence	featureprop	Value	published_symbol	In-house	Published QTL symbol.
Bayes factor	Sequence	featureprop	Value	bayes_factor	In-house	Bayes factor as an evidence of the reported QTL
*P* values	Sequence	featureprop	Value	P_value	In-house	p values as an evidence of the reported QTL
R square	Sequence	featureprop	Value	R_square	In-house	The percentage of the total genetic variance explained by the locus
LOD	Sequence	featureprop	Value	LOD	In-house	LOD value as an evidence of the reported QTL
Additive effect	Sequence	featureprop	Value	additive_effect	In-house	Additive effect of the QTL allele
dominance effect	Sequence	featureprop	Value	dominance_effect	In-house	Dominance effect of the QTL allele
Direction	Sequence	featureprop	Value	direction	In-house	direction of the QTL effect
Screening method	Sequence	featureprop	Value	screening_method	In-house	Any screening method for the phenotyping
Comments	Sequence	featureprop	Value	comments	In-house	Any comments
Other data linked to QTL						
Data type	Chado module^a^	Table name	Linking table^c^	Description		

Trait name	Sequence	cvterm	feature_cvterm	Trait Ontology term that is associated with the QTL		
Alias	Sequence	synonym	feature_synonym	Any alias for the QTL		
Source	Stock	stock	feature_stock	Parent germplasm with the desirable allele		
Reference	Pub	pub	feature_pub	Associates a publication stored in the pub table with the QTL		
Dataset	Project	project	feature_project*	Dataset that includes all the QTL reported in the publication		
Contact	Contact	contact	feature_contact*	The individual that submitted the QTL has a record in the contact table and is associated with the QTL		
Colocalized marker	Sequence	feature	feature_relationship	Colocalized marker is a separate record in the feature table (with type_id as SO:genetic_marker). The relationship type_id 'located_in', the colocalized marker is the subject_id and the QTL is the object_id		
Neighboring marker	Sequence	feature	feature_relationship	Neighboring marker is a separate record in the feature table (with type_id as SO:genetic_marker). The relationship type_id 'adjacent_to', the neighboring marker is the subject_id and the QTL is the object_id		

a The Chado modules are for Chado version 1.2.

b The vocabularies are: Sequence Ontology (SO), In-House (term add to the GDR/CottonGen internal vocabularies).

c Tables with an asterisk (*) are custom tables.

The mean trait values of each parent and progeny population are stored in the phenotype table and linked to the stock table through the nd_experiment table. Each row of the nd_experiment table is associated to the project record that describes the entire QTL dataset via the nd_experiment_project table. The statistical values for the trait measurements such as standard deviation, coefficient of variation and skewness, are stored in the nd_experimentprop table (Figure 2, Supplementary File S1). When the same trait was measured in multiple years, in multiple sites, and/or in multiple environmental conditions, these site/environment data are stored in the nd_geolocation table, which is linked to the specific trait measurement for a stock via the nd_experiment table. 

### MTL

Data of monogenic traits, which follows Mendelian inheritance pattern are stored in the feature table with the SO term ‘heritable_phenotypic_marker’ as the type (Figure 1; Table 5). The name, alias, published symbol, associated molecular markers are stored in the same way as QTL. MTL are also associated with TO terms via the feature_cvterm table. The positions of the MTL in genetic maps are stored the same way as genetic markers and QTL (Figure 2). If the underlying genes for the MTL are known, the relationship is stored using the feature_relationship table with the SO term ‘associated_with’ as the type.

**Table 5.** Storage of MTL in Chado. MTL are stored in feature table with a type_id of 'heritable_morphological_marker'

Data type	Chado module^a^	Table name	Field name	Vocabulary terms for type_id	Vocabulary^b^	Description
MTL name	Sequence	feature	uniquename			curator-defined label for MTL
organism	Sequence	feature	organism_id			The organism to which the MTL belongs. A foreign key to the organism table
type	Sequence	feature	type_id	heritable_morphological_marker	SO	All MTL are of the SO type: 'heritable_morphological_marker'
Properties of MTL						
Data type	Chado module^a^	Table name	Field name	Vocabulary terms for type_id	Vocabulary^b^	Description

Published symbol	Sequence	featureprop	value	published_symbol	In-house	Published MTL symbol
Screening method	Sequence	featureprop	value	screening_method	In-house	Any screening method for the phenotyping
Description	Sequence	featureprop	value	description	In-house	Any description on the MTL
Comments	Sequence	featureprop	value	comments	In-house	Any comments
Other data linked to MTL						
Data type	Chado module^a^	Table name	Linking table^c^	Description		

Trait name	Sequence	cvterm	feature_cvterm	Trait Ontology term that is associated with the MTL		
Alias	Sequence	synonym	feature_synonym	Any alias for the MTL		
Source	Stock	stock	feature_stock	Parent germplasm with the desirable allele		
Reference	Pub	pub	feature_pub	Associates a publication stored in the pub table with the QTL		
Dataset	Project	project	feature_project*	Dataset that includes all the QTL reported in the publication		
Contact	Contact	contact	feature_contact*	The individual that submitted the QTL has a record in the contact table and is associated with the QTL		
Colocalized marker	Sequence	feature	feature_relationship	Colocalized marker is a separate record in the feature table (with type_id as SO:genetic_marker). The relationship type_id 'located_in', the colocalized marker is the subject_id and the QTL is the object_id		
Neighboring marker	Sequence	feature	feature_relationship	Neighboring marker is a separate record in the feature table (with type_id as SO:genetic_marker). The relationship type_id 'adjacent_to', the neighboring marker is the subject_id and the QTL is the object_id		

a The Chado modules are for Chado version 1.2.

b The vocabularies are: Sequence Ontology (SO), In-House (term add to the GDR/CottonGen internal vocabularies).

c Tables with an asterisk (*) are custom tables.

## Germplasm data

### Germplasm detail and pedigree data

**Figure 3:**
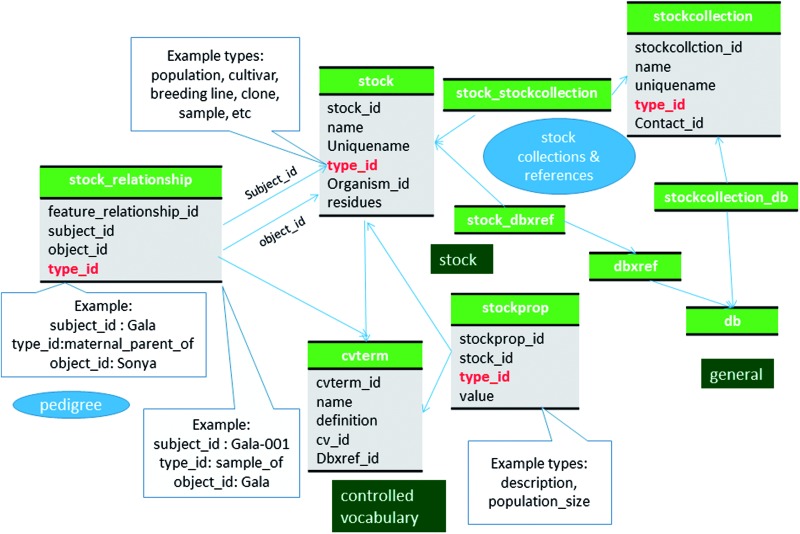
Schematic diagram of how stocks are stored in Chado. Hierarchical stocks, from samples, cultivars to population are stored in samples and their relationship including pedigree are stored in stock_relationship table. The bold red fields represent foreign keys to the cvterm table which houses vocabulary terms. Boxes in dark green represents the modules of Chado represented in this diagram.

Germplasm data that are associated with genomic (including source germplasm of marker sequences) and genetic data, as well as those used in large scale phenotypic and genotypic experiments, such as cultivars and breeding lines are stored in the stock table (Figure 1). The mapping populations and parents used for genetic mapping and QTL analyses are also stored in the stock table (Figure 2). The stock.type_id field is used to identify the type of record in the stock table. However, there is no appropriate vocabulary with terms that encompasses all of the data for this table. Therefore, the vocabulary terms used to identify records in the stock table are part of an in-house vocabulary that includes terms such as ‘cultivar’, ‘breeding/research material’ or ‘wild/unimproved’, for the germplasm type. The term ‘population’ is used as the type for population records such as for mapping populations. Pedigree data are stored using the stock_relationship table (Figure 3) and vocabulary terms such as ‘paternal_parent_of’, ‘maternal_parent_of’, ‘mutational_parent_of’ are used in the stock_relationship.type_id field and are used to define the pedigree relationship between two germplasm. Ancillary data for each germplasm, such as aliases and descriptions are stored in the stockprop table (Supplementary File S1). When the images of stocks are available, we store the image in the eimage table and link to the feature table using the stock_image table (Supplementary File S1). The title, description and the person who provided the image are stored as described above.

### Clones and samples

In addition to cultivars or breeding lines with a distinct genotype, samples for a particular phenotyping experiment, such as a group of peach fruits collected at a certain date, are also stored in the stock table. Entries in the stock table therefore include hierarchical entities such as a population, cultivars, breeding lines, clones or samples (Figure 3). For example, if phenotype measurements are available for fruits collected from different individual trees two times a year, a distinct record is added to the stock table with a unique sample ID (used as the stock.uniquename) and represents the fruit samples collected each time. This ‘sample’ stock record is associated to the phenotypic value stored in the phenotype module through the nd_experiment table as previously described (18). The relationship between these ‘sample’ records and the germplasm are defined in the stock_relationship table with the term ‘sample_of’. If multiple clones of the same cultivar is planted, those clones are stored in the stock table and are linked to the cultivar using the stock_relationship table with the term ‘clone_of’.

### Stock center data

In CottonGen, information about stock centers and their available stock collections are stored using the stockcollection table and the db table (Figure 3, Supplementary File S1). Each stock collection has a unique code assigned by CottonGen curators which is stored in the stockcollection.uniquename and db.name fields. The two tables are linked by a custom table named stockcollection_db. The real collection name is stored as stockcollection.name and the description of the stock center is stored in db.description. The web address (URL) of the stock center is stored in the db.url field. The unique accession ID of each stock in the collection is stored in the dbxref.accession field and is also used as the name of the stock in the stock.name field. The stocks in a collection are linked to the stock collection via the the stock_dbxref and stockcollection_stock tables.

### Passport data

**Figure 4:**
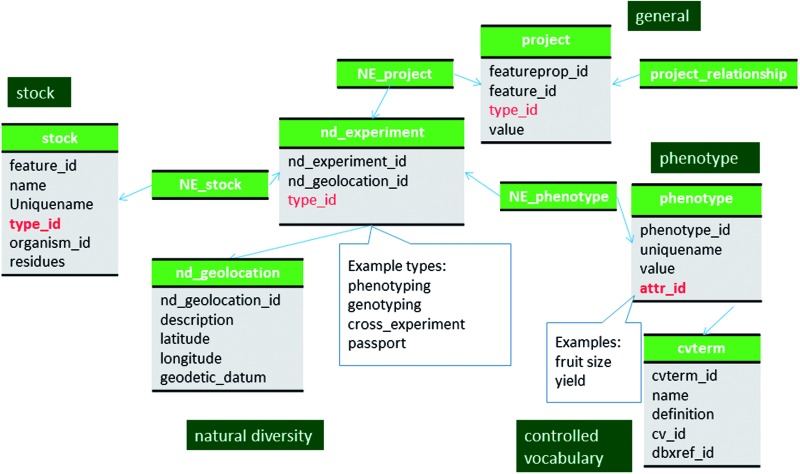
Schematic diagram of how phenotypic data are stored in Chado. Datasets, such as passport data and cross data, which do not have associated phenotypic or genotypic data can also be stored in the nd_experiment table and linked to the stock table. The bold red fields represent foreign keys to the cvterm table which houses vocabulary terms. Boxes in dark green represents the modules of Chado represented in this diagram.

In CottonGen, the passport information, which can include the collector, location, environmental condition of the original germplasm collection, is also stored using the tables in the modules of natural diversity, stock and contact (Figure 4). A record is created in the nd_experiment table with the in-house defined term ‘passport’ as the type_id. Information about the physical location of the germplasm is stored in nd_geolocation data and details of the original collection such as population size, sample size and comments are stored in the nd_experimentprop table. The collector information is stored in the contact table and linked by the nd_experiment_contact.

## Phenotypic data

Large-scale phenotypic data from projects such as germplasm evaluation and breeding are stored as unique records in the project table of Chado. When a project is composed of several smaller projects, such as datasets from different years, location and environmental conditions, the smaller project can be linked to a larger project using the project_relationship table. The natural diversity module is then used to associate phenotypic data from those studies (Figure 4). A record is created in the nd_experiment table with the in-house defined term ‘phenotyping’ used in the nd_experiment.type_id field for each phenotypic measurement from a specific sample. As prescribed in the previous publication (18) each measurement is given a unique record in the nd_experiment table. The record in the nd_experiment table is linked to the stock table via the nd_experiment _stock table. Hence the nd_experiment table links the stock sample in the stock table and the phenotype record stored in the phenotype table.

**Figure 5:**
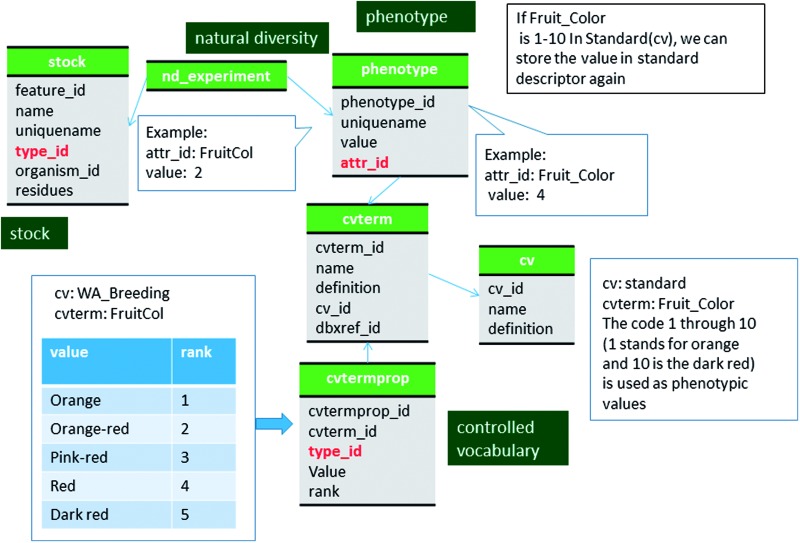
Schematic diagram of how coded phenotypic values are stored in Chado. The same data can be stored in two different code system to enable comparison among datasets. The bold red fields represent foreign keys to the cvterm table which houses vocabulary terms. Boxes in dark green represents the modules of Chado represented in this diagram.

Measurements from the same project are associated to a project using the nd_experiment_project table. The phenotype table stores a specific value (phenotype.value) for a specific trait descriptor (phenotype.attr_id) as distinct records. The trait descriptor and the value, concatenated by an underscore, serve as the uniquename for a phenotype and is stored in the phenotype.uniquename field of the phenotype table. When the trait descriptors are qualitative and therefore have numeric codes, each code is stored in cvtermprop table using the value and rank fields and linked to the term to which they belong (Figure 5). A set of trait descriptors from the same project belong to the same entry in the cv table. Depending on the projects, the same trait can be measured by two different trait descriptors with different coding system. For example, fruit color can be recorded using the code system of one through five; one for the lightest and five for the darkest red color. Another project may record fruit color using the code system from one through ten. If the community select the coding system from one through ten as the standard, it is possible to store the fruit color using both trait descriptors, the original and the standard. This will allow comparison of data from projects using different coding systems (Figure 5). It is not yet implemented in GDR or CottonGen but it will be possible to store the same data using multiple trait descriptors if a standard descriptor set is developed by the community.

## Genotypic data

**Figure 6:**
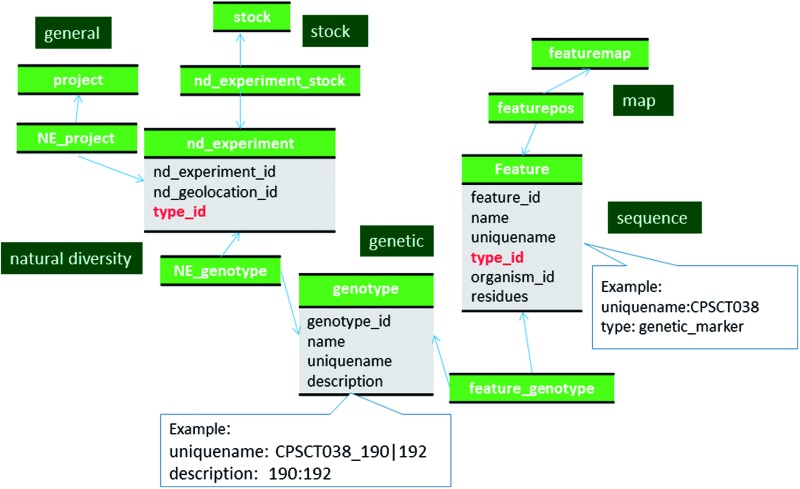
Schematic diagram of how genotypic data are stored in Chado. The bold red fields represent foreign keys to the cvterm table which houses vocabulary terms. Boxes in dark green represents the modules of Chado represented in this diagram.

Large scale genotypic data from projects such as germplasm evaluation and breeding are also stored using the project, genotype and natural diversity modules of Chado similar to phenotypic data (Figure 6, Supplementary File S1). A specific genotype for a specific marker is stored as a distinct record in the genotype table. We store genotype as a concatenation of alleles, in the genotype.description field in order to store allele copy number. The alleles for a genotype are alphanumerically ordered and each allele is separated by a bar character, ‘|’. The marker used for genotyping is stored as a record in the feature table as described previously for genetic markers, and linked to the genotype table using the feature_genotype table. The marker name and the genotype, concatenated by an underscore, is used for the genotype.uniquename field. In addition to marker alleles, haplotype of a genomic region and MTL are also stored in the genotype table in the same way as the marker alleles (Figure 6). Haplotype represents a unique set of marker alleles in adjacent physical genomic locations. Haplotype blocks are stored in the feature table using the SO term ‘haplotype_block’ and the relationship between the haplotype block and the markers within the block is stored in the feature_relationship table with the SO term ‘contains’.

A record is created in the nd_experiment table with type ‘genotyping’ for each genotypic measurement on a specific record of the stock table (such as a cultivar, breeding or research material). The row in the nd_experiment table is linked to the stock table via the nd_experiment_stock table. Hence, the nd_experiment table links the stock sample in the stock table and the genotype record stored in the genotype table.

As with phenotypic data, measurements from a single project are linked to appropriate record in the project table. If one funded project produced multiple genotyping experiments on different sets of stocks (e.g. stocks of different species and/or maintained by different breeders), a project can be created for each set and then the small projects can be linked to a larger project (e.g. funded project) by the project_relationship table.

## Relationship between genotype and phenotype

**Figure 7:**
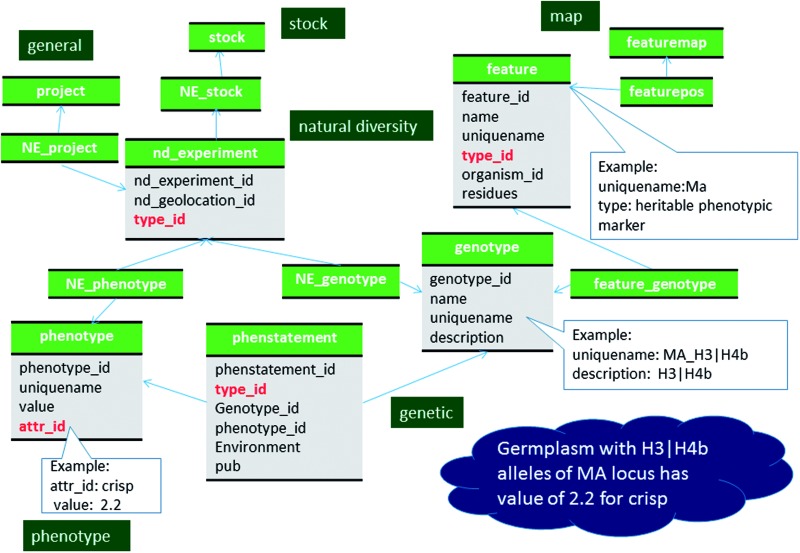
Schematic diagram of how relationship between genotype and phenotype is stored in Chado. The bold red fields represent foreign keys to the cvterm table which houses vocabulary terms. Boxes in dark green represents the modules of Chado represented in this diagram.

Some breeding projects, such as RosBreed (33), provide data for each haplotype’s effect on a specific trait as phenotypic values when such data are available. The phenotypic values are stored in the phenotype table and the haplotypes are stored in the genotype table as stated above. The relationship between haplotypes and haplotype effects are stored using the phenstatement table (Figure 7, Supplementary File S1).

## Custom tables and ontologies

To support storage of all the data types discussed, a set of custom tables is needed. These custom tables adhere to the typical design of existing Chado tables and are primarily of three types: linker tables, property tables and relationship tables. Linker tables are used to associate two different data types, property tables are used to associate ancillary data with a specific record, and relationship tables indicate relationships between data. The set of custom tables are: eimageprop, eimage_contact, feature_image, stock_image, organism_image, featuremapprop, featuremap_stock, featuremap_organism (to link map data to parents and mapping population), feature_project (some features like QTL needs to be linked to a dataset), organism_relationship (to record relationships between organisms like 'fertile_with', 'steril_with', and 'incompatible_with'), stockcollection_db (for linking stock collections stored in the stockcollection table and their external database records stored in db table), feature_stock, feature_contact, library_stock, library_contact, contactprop, featuremap_contact, featuremap_dbxref, featureposprop, organismprop, analysis_organism and pubauthor_contact (for associating features, stocks, contacts, libraries, and their properties). Fortunately, these custom tables have been reviewed by the Chado community and will be included in the forthcoming Chado v1.3 release. The SQL statements to create these custom tables can be found under each Chado module in the GitHub repository (https://github.com/GMOD/Chado/tree/1.31/chado/modules). We use Sequence Ontology, Gene Ontology and Trait Ontology to describe the feature type and relationship, gene and trait, respectively. For others we use custom developed vocabularies as described in the text and the tables.

## Conclusion

The sequence module is central for the storage of sequence features, their relationships and has the most well-defined use cases for Chado as described in detail by Mungall *et al.* (1). Large-scale phenotypic and genotypic data and their experimental details can be stored using the newly added Natural Diversity module and the pre-existing stock, genetic and phenotype modules. The use cases for how to store these data has also been described in detail (18). This manuscript provides an important use case for how genomic data, large scale phenotypic and genotypic data can be integrated and stored with other data such as QTL and genetic mapping data. In summary, genomic features without sequences, such as linkage groups, bins, QTLs and heritable phenotypic markers, can be stored in the feature table and their genetic map position can be stored using the map module. Genetic markers that are within or near QTL can be stored using the feature_relationship table. When underlying genes for QTL are identified, the relationship can also be stored in the feature_relationship table. Phenotypic descriptors used for cultivar evaluation or breeding projects as well as QTL can be stored using Trait Ontology, allowing users to view all the phenotypic data, QTL and germplasm that are associated with a specific trait ontology term. The genetic module can be used to store data from large scale genotypic data, with natural diversity and stock modules to store project details and associated germplasm. The SNPs or haplotype blocks can be stored in the feature table, linked to the genotype table which stores alleles, genotype (allelic combination for the locus) or both, depending on the project requirements. The relationship between genotype and phenotype, including numerical values such as haplotype effect, can be stored in the phenstatement table in the genetic module. With some custom tables, we found that Chado can accommodate all our genomic, genetic and breeding data for Rosaceae and CottonGen.

GDR and CottonGen are built using Tripal which uses Chado as the data storage back-end. The adoption of Chado in the development of databases has increased greatly due to the availability of Tripal. With the support of federal granting agencies such as the USDA and NSF for the adoption and further development of Tripal, databases built using both Chado and Tripal are expected to grow in the near future. Thus, we expect the information provided here should assist those adopting Chado and Tripal to plan and implement storage of the types of data described here.

## Supplementary data

Supplementary data are available at Database Online. 

*Conflict of interest*. None declared.

Supplementary Data
