# Chado use case: storing genomic, genetic and breeding data of Rosaceae and Gossypium crops in Chado

**DOI:** 10.1093/database/baw058

**Published:** 2016-05-02

**Authors:** Sook Jung, Taein Lee, Stephen Ficklin, Jing Yu, Chun-Huai Cheng, Dorrie Main

doi:10.1093/database/baw010

In Table 1 of this article, the description for Data Type: Marker locus should have read as follows:

When marker is localized in genetic maps, the locus is stored separately in the feature table (with type_id as SO:marker_locus). The relationship type_id is ‘instance_of’, the subject_id is the locus and the object_id is the marker.

The publisher apologizes for this error.

