# Supplementary material for: Chado use case: storing genomic, genetic and breeding data of Rosaceae and Gossypium crops in Chado
Source: Database (Oxford). 2016 Mar 17;2016:baw010. doi: 10.1093/database/baw010 (PMC4795932; doi:10.1093/database/baw010)
Supplement: Supplementary Data [file supp_2016_baw010_index.html]

Chado use case: storing genomic, genetic and breeding data of Rosaceae and Gossypium crops in Chado — Supplementary Data 

# Chado use case: storing genomic, genetic and breeding data of Rosaceae and Gossypium crops in Chado

## Supplementary Data

files

- Supplementary Data - zip file
