# Supplementary figures and images for: Chado use case: storing genomic, genetic and breeding data of Rosaceae and Gossypium crops in Chado
Source: Database (Oxford). 2016 Mar 17;2016:baw010. doi: 10.1093/database/baw010 (PMC4795932; doi:10.1093/database/baw010)

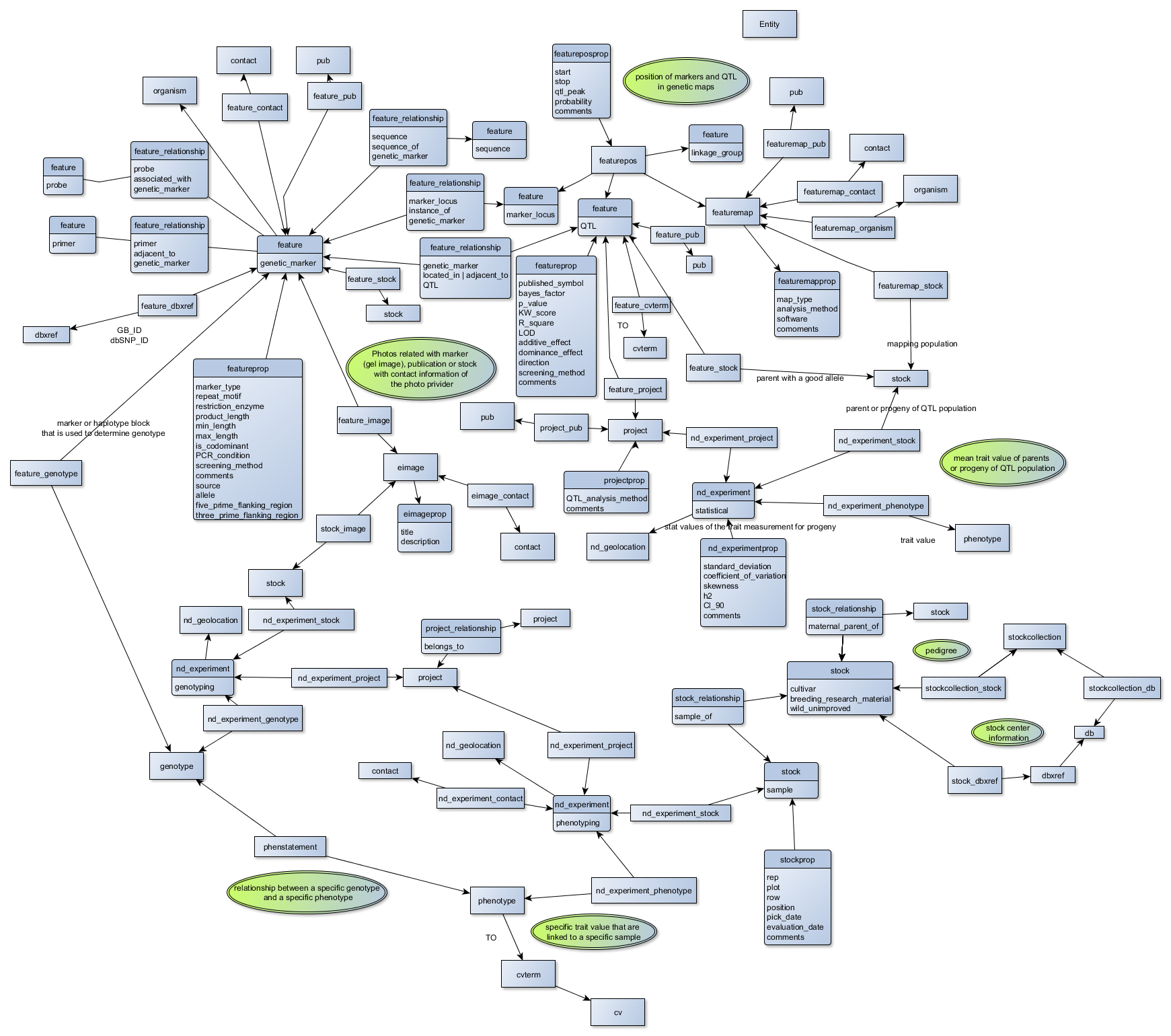

Supplement: Supplementary Data [file supp_baw010_suppl_data.zip › GDR_CottonGEN_chado-supple.png]
